# An Enhanced Dynamic Simulation Model of a Hybrid Magnetic Bearing Taking Account of the Sensor Noise

**DOI:** 10.3390/s20041116

**Published:** 2020-02-18

**Authors:** Dawid Wajnert, Jan K. Sykulski, Bronislaw Tomczuk

**Affiliations:** 1 Department of Electrical Engineering and Mechatronics, Opole University of Technology, PL-45758 Opole, Poland; d.wajnert@po.edu.pl; 2 Electronics and Computer Science, University of Southampton, Southampton, SO17 1BJ, UK; jks@soton.ac.uk

**Keywords:** sensor noise, hybrid magnetic bearing, simulation model, nonlinear magnetic equivalent circuit

## Abstract

The paper presents simulation and experimental verification of the hybrid magnetic bearing (HMB) performance characteristics. It has been demonstrated that the additional errors from the eddy current sensors have a significant impact on the control signals. An improved mathematical model combines a nonlinear magnetic equivalent circuit of the HMB with the ordinary differential equations of its transients. These equations describe the rotor motion and the electric circuit of the system, as well as the control system required for stable levitation of the rotor. Certain harmonics have been observed in the displacement signals of frequencies equal to the multiples of the cylinder rotations. The calculation model has, therefore, been improved, taking into account the interference of the harmonics. Simulation results were validated by comparing the time responses of the transients obtained from the numerical calculations with those measured on a real object; a satisfactory agreement between the results has been achieved.

## 1. Introduction

Investigations of transients in magnetic bearings require a computationally efficient simulation model that encompasses the control system. Several simulation models dedicated to the calculation of transients in electrical machines have been developed. The first type is a field-circuit directly coupled finite element model (FEM), which combines magnetic field equations with equations of the external circuit and mechanical motion [1]. In this model, the flux linkages––as well as the electromagnetic torque or force––are computed using field quantities. Both 2D and 3D magnetic field formulations with nonlinear B-H characteristic can be implemented to simulate magnetic field distributions [2,3,4,5].

Eddy currents and hysteresis of the magnetic material can also be incorporated into magnetic field equations [6,7]. Additionally, the circuit equations can assimilate an inverter supply system [3], the distorted voltage-excited sources [8], as well as those from capacitors [9]. Moreover, this type of model facilitates coupling between the electromagnetic and thermal fields [10]. Contemporary software dedicated to the calculation of magnetic field distributions (e.g., Ansys Maxwell 3D, Comsol) allows performing the simulations of transients based on the field-circuit directly coupled FEM [4]; it is even possible to incorporate (into the simulation model) the required control loops essential to achieve magnetic levitation [7]. Unfortunately, this model suffers from relatively long execution times and significant computation effort, as magnetic field equations need to be solved at every simulation step of the circuit and mechanical motion equations.

The second type of simulation model is a field-circuit indirectly coupled FEM [1]. For this model, the flux linkages—as well as the electromagnetic torque or force—are calculated a priori based on the magnetic field model. Calculation results are then incorporated into the circuit and mechanical motion equations as look-up tables [11,12,13,14,15]. This model facilitates the implementation of the control loops as well as the pulse width modulation inverter and supply from the capacitor banks. It is characterized by reasonable accuracy of the results as well as relatively short calculation times.

Another type of simulation model not yet mentioned is a field-circuit directly coupled magnetic equivalent circuit model. The electromagnetic torque, or force, and the flux linkages of the electrical machine are calculated from the magnetic equivalent circuit (MEC) at every solution step when solving the electric and motion equations [16]. The MEC of the electric machine can be constructed by applying a circuit approach [17,18,19] or by using the edge elements equations [20,21,22]. The MEC of an electric machine can incorporate nonlinear characteristics of the magnetic material as well as leakage and fringing fluxes [23]. The field-circuit directly coupled magnetic equivalent circuit also facilitates the implementation of the control loops [16]. For the field-circuit directly coupled FEM, differential equations of the machine (equations of the electric circuits and motion) are substituted with algebraic equations for subsequent time steps. Therefore, the magnetic field, current propagation, and motion are solved simultaneously, often with the aid of the Newton-Raphson iterative procedure due to the nonlinear characteristic of the magnetic material [8]. The magnetic field is solved using the finite element method. However, the simulation model presented in this research uses a nonlinear magnetic equivalent circuit model that is solved independently from the differential equations of the machine and the results from the MEC model are forwarded to the equations of the electric circuit and motion for subsequent time steps. A significant advantage of this simulation model in relation to the field-circuit directly coupled FEM is fast calculation times and low computational cost.

Unfortunately, the results obtained from simulation models are often significantly different from those collected by measurements [24]. One of the reasons behind such discrepancies is not so much related to the imperfections of the models but is due to errors caused by the measurement system. Thus, to improve the overall accuracy of the simulations, errors in measurements should also be accounted for. The aim of this research is to formulate a simulation model of an active magnetic bearing with permanent magnets dedicated to the simulation of transients; the model will be based on the field-circuit directly coupled magnetic equivalent circuit and will embrace measurement errors generated by the eddy current proximity sensors.

## 2. Description of the Hybrid Magnetic Bearing

**Figure 1:**
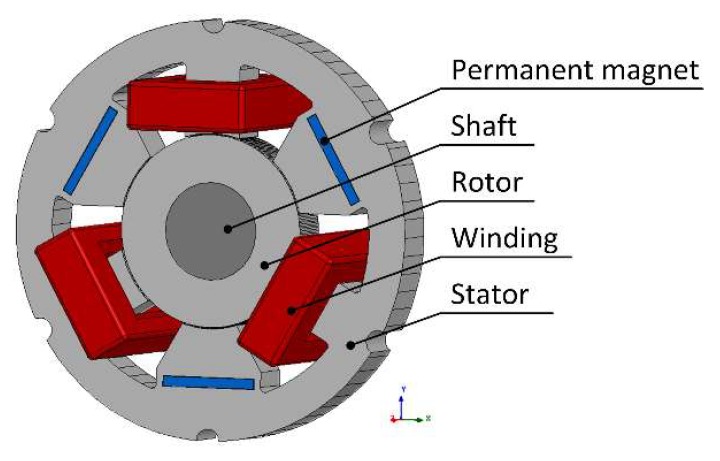
The geometry of the hybrid magnetic bearing (HMB).

**Figure 2:**
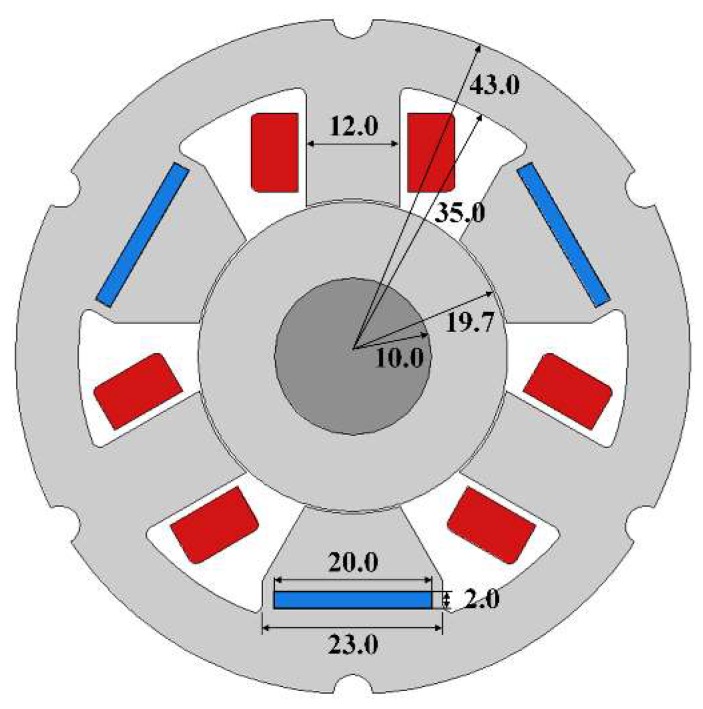
The main dimensions of the HMB.

Figure 1 depicts a 6-pole radial magnetic bearing with permanent magnets, also known as a hybrid magnetic bearing (HMB). Three permanent magnets N38 are installed in holes cut from poles, to provide bias flux. The permanent magnets are magnetized along the shortest edge in order to generate magnetic flux towards the rotor. Each winding contains 100 turns. The geometric air gap between the stator and the rotor equals 0.3 mm. Figure 2 shows the main dimensions of the HMB; the axial length of the HMB stator is equal to 10 mm. All poles of the HMB stator generate magnetic forces due to the magnetic flux in the air gap of the ferromagnetic circuit. The three poles with windings can control the value of the magnetic force along the three axes. The main parameters of the HMB are listed in Table 1.

## 3. The dynamic simulation model of the HMB

### 3.1. Equations for the Dynamic Simulation Model

The field-circuit directly coupled magnetic equivalent model of the HMB consists of three components. The first part constitutes the equations that describe electrical circuits and mechanical motion of the rotor. The following equations describe the voltages across the windings of the HMB,

(1) $$u_{1}=R_{1}i_{1}+\frac{d\Psi_{1}\left(x,y,i_{1},i_{2},i_{3}\right)}{dt}$$

(2) $$u_{2}=R_{2}i_{2}+\frac{d\Psi_{2}\left(x,y,i_{1},i_{2},i_{3}\right)}{dt}$$

(3) $$u_{3}=R_{3}i_{3}+\frac{d\Psi_{3}\left(x,y,i_{1},i_{2},i_{3}\right)}{dt}$$

where *u*_1_, *u*_2_, *u*_3_ denote supplying voltages, *R*_1_, *R*_2_, *R*_3_ indicate the resistances of the windings, *i*_1_, *i*_2_, *i*_3_ describe the current densities excited in the windings, while *Ψ*_1_, *Ψ*_2_, *Ψ*_3_ are the flux linkages for the three windings. The motion of the HMB rotor is described by the following equations

(4) $$m\frac{dx^{2}}{dt^{2}}=F_{x}\left(x,y,i_{1},i_{2},i_{3}\right)+m\omega^{2}e_{s}cos\left(\omega t\right)$$

(5) $$m\frac{dy^{2}}{dt^{2}}=F_{y}\left(x,y,i_{1},i_{2},i_{3}\right)-mg+m\omega^{2}e_{s}sin\left(\omega t\right)$$

where *F_x_* and *F_y_* denote the magnetic forces acting along the *x*- and *y*-axis, respectively. The symbol *m* indicates the mass of the rotor, *g* is the gravitational acceleration, *e_s_* describes the eccentricity of the rotor, and *ω* denotes the angular velocity of the rotor. Equations (4) and (5) include the gravity force, and the static unbalance force. The equations of motion presented above describe the dynamics of a rigid rotor because the maximal rotating speed is lower than the first critical speed of the shaft. Moreover, the rotor dynamics omits the gyroscopic effect because the diameter of the rotor is much smaller than its length, and the assumed rotating speed of the rotor is not very high.

**Figure 3:**
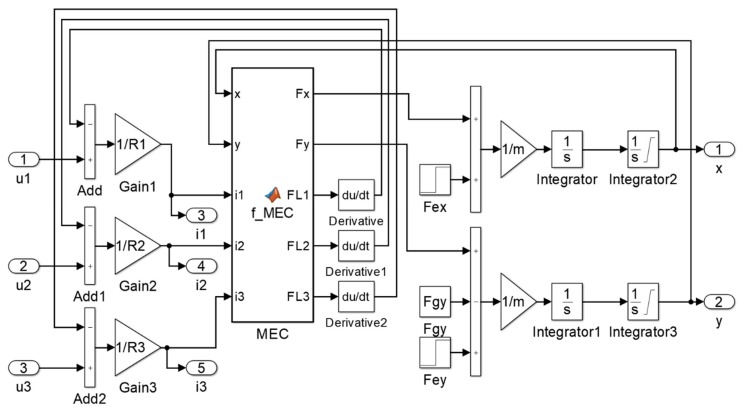
Implementation of the HMB simulation model in MATLAB/Simulink.

Figure 3 presents the implementation of the HMB simulation model in MATLAB/Simulink (MathWorks Ltd., Natick, USA), according to Equations (1–5). The magnetic forces *F_x_* and *F_y_*, as well as the flux linkages *Ψ*_1_, *Ψ*_2_, *Ψ*_3_ are calculated using the MEC with HMB incorporated into the simulation model as a separate block. This simulation model does not take into account the errors of the position measurement.

The constant parameters of the simulation model are listed in Table 2.

### 3.2. The Nonlinear Magnetic Equivalent Circuit of the HMB

**Figure 4:**
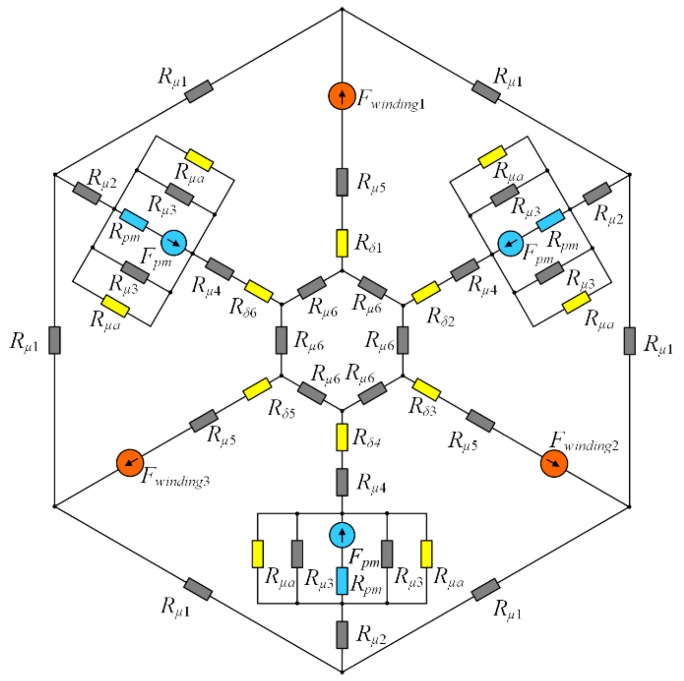
A diagram of the magnetic equivalent circuit for the HMB.

Figure 4 shows a diagram of the MEC of the HMB that constitutes the second component of the dynamic simulation model.

Due to the magnetic nonlinearity of the stator and rotor materials, the reluctances (*R_μ_*_1_ to *R_μ_*_6_) of the magnetic fluxes are nonlinear; their values depend on branch fluxes. The reluctances (*R_δ_*_1_ to *R_δ_*_6_) of the air gap paths between the stator and rotor include the fringing flux by the application of Carter’s coefficient calculated from 3D finite element analysis [25]. The MEC of the HMB also includes the reluctances of the permanent magnets (*R_pm_*) and the reluctances of the leakage flux paths in the proximity of the permanent magnets (*R_μa_*). Kirchhoff’s laws for magnetic circuits were used for derivation of a system of nonlinear equations for the branch fluxes

(6) $$f\left(\varphi \right)=A_{R}\left(\varphi \right)\varphi -F=0$$

where **A_R_** is the matrix of reluctance elements, **φ** denotes a vector with the unknown magnetic branch fluxes, while **F** indicates the vector of magnetomotive forces generated by windings and permanent magnets. The system of 30 nonlinear equations (6) is solved by the iterative Broyden’s method [26].

The magnetic forces *F_x_* and *F_y_* were calculated according to the following expressions

(7) $$F_{x}=\frac{\sqrt{3}}{2}F_{2}+\frac{\sqrt{3}}{2}F_{3}-\frac{\sqrt{3}}{2}F_{5}-\frac{\sqrt{3}}{2}F_{6}$$

(8) $$F_{y}=F_{1}+\frac{1}{2}F_{2}-\frac{1}{2}F_{3}-F_{4}-\frac{1}{2}F_{5}+\frac{1}{2}F_{6}$$

where the magnetic force generated by each pole *F_i_* was calculated from the expression

(9) $$F_{i}=k_{c}\frac{\phi_{i}^{2}}{2{µ}_{0}A_{AirGap}}$$

where *k_c_* denotes Carter’s coefficient, *ϕ_i_* stands for the magnetic flux flowing through the pole *i*∈〈1,6〉, *μ*_0_ means the magnetic permeability of free space, *A_AirGap_* denotes a cross-sectional area of the pole magnetic flux in the air gap.

Flux linkages *Ψ*_1_, *Ψ*_2_, *Ψ*_3_ are calculated from the magnetic fluxes multiplied by the turns number *N*. The full description of the MEC for the HMB and its validation may be found in [25].

### 3.3. Description of the Control System

All hybrid magnetic bearings, similar to active magnetic bearings, are unstable devices, therefore, they require control systems for their operation. The most widely used control strategy in industrial magnetic bearing systems is the current control scheme [27]. It involves a control system that has two control loops: Underlying and overlying. The underlying loops regulate the windings currents *i*_1_, *i*_2_, *i*_3_ through the adjustment of the pulse with modulation signal in the switching power amplifiers. The overlying loops regulate the position of the rotor through a change of control currents. For the HMB under consideration, the position of the rotor can be controlled in two or three axes. The control of the rotor position in the two axes requires only two proximity sensors and two position controllers. Due to simplicity, this control method was used in this work. Consequently, two control currents *i_x_* and *i_y_* have to be converted into winding currents *i*_1_, *i*_2_ and *i*_3_ as

(10) $$i_{1}=i_{y}$$

(11) $$i_{2}=-\frac{1}{2}i_{y}+\frac{\sqrt{3}}{2}i_{x}$$

(12) $$i_{3}=-\frac{\;1}{2}i_{y}+\frac{\;\sqrt{3}}{2}i_{x}$$

Control methods like proportional-derivative (PD), proportional-integral-derivative (PID), robust control, linear-quadratic (LQ) can be used to control the position of the rotor [27,28,29,30]. However, the most commonly used type of controller is PID, as it is simple and effective. The position of the rotor in axes *x* and *y* are controlled by two discrete PID controllers

(13) $$G_{PID}(z)=K_{P}+K_{I}\frac{T_{s}}{z-1}+K_{D}\frac{N}{1+\frac{T_{s}N}{z-1}}$$

where *K_P_*, *K_I_* and *K_D_* are the parameters of the controller, *T_s_* denotes the sampling time and *N* indicates the filter coefficient of the derivative.

The windings currents *i*_1_, *i*_2_, *i*_3_ are controlled by three discrete proportional-integral (PI) controllers

(14) $$G_{PI}\left(z\right)=K_{P}+K_{I}\frac{T_{s}}{z-1}$$

The parameters of the position for the PID controllers were calculated according to the method presented in reference [31] and are listed in Table 3.

The parameters of the proportional-integral (PI) current controllers were obtained by a manual adjustment in order to obtain a fast response and acceptable overshooting. Their values are presented in Table 4. All controllers have the integrator anti-windup circuits included [32].

### 3.4. Implementation of the Dynamic Simulation Model

**Figure 5:**
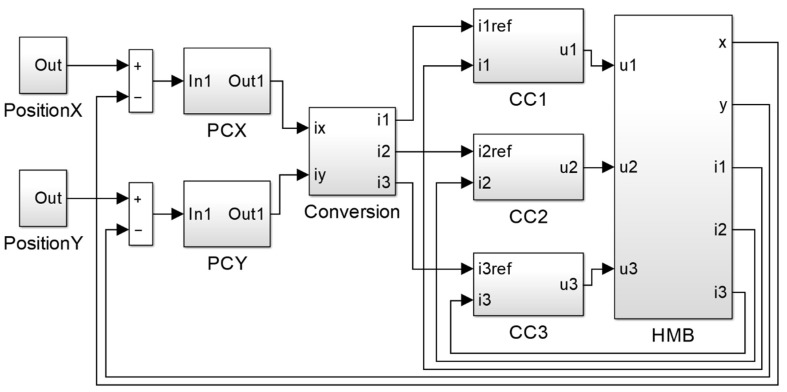
Implementation of the dynamic simulation model in MATLAB/Simulink.

Figure 5 depicts the implementation of the dynamic control system in MATLAB/Simulink; its abbreviations denote PCX—position controller in the *x*-axis, PCY—position controller in the *y*-axis, CC1, CC2, CC3—current controllers in the windings 1, 2, and 3. The block “Conversion” implements Equations (10–12). Two blocks, “PositionX” and “PositionY” are responsible for the generation of the required rotor position.

The transients were simulated using a fixed-step Euler’s procedure with a step size of 50 μs. The system of equations for the MEC in the HMB has been solved for each time step of the dynamic simulation. The execution time for a transient of the duration of 1 s was about 300 s of central processing unit (CPU) operation for a computer with Intel Xeon E5-1650 3.5 GHz processor and installed 16 GB RAM.

## 4. Simulation Results and Measurements

Measurement tests were performed on a real object to verify the correctness and accuracy of the proposed simulation model.

**Figure 6:**
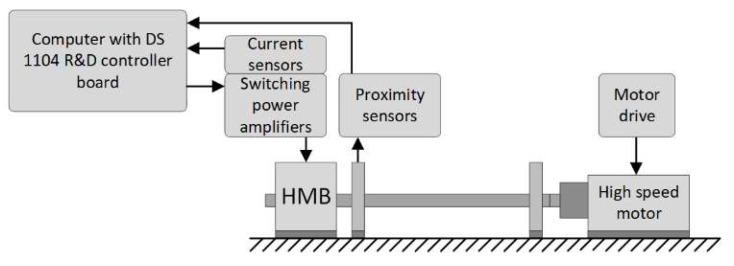
A test bench for the HMB.

Figure 6 depicts a test bench that consists of the HMB actuator, a drive with a high-speed motor, two proximity sensors, three switching power amplifiers with current sensors, and a computer with a DS 1104 R&D controller board (dSPACE GmbH, Paderborn, Germany). The position of the rotor in the *x* and *y* axes was measured by two eddy current proximity sensors MDS10/MDT10 (Technicad Ltd., Gliwice, Poland). Their measurement range is from 0.5 mm to 2.5 mm, and the frequency response 0÷10 kHz. The switching power amplifiers operate in a full H-bridge configuration with frequency 50 kHz. The current was measured using three LEM LTS-6NP sensors (LEM Holding SA, Fribourg, Switzerland). The controller board DS1104 performed the following tasks: Analog to digital conversion of the current and position signals, execution of three PI current controllers, and two PID position controllers as well as generation of the pulse width modulation (PWM) signals for switching the power amplifiers. The current signals were converted with a frequency of 20 kHz at the resolution of 1.693 mA/bit. The position signals were converted with a frequency of 10 kHz at the resolution of 0.03815 μm/bit. A full description of the test bench and its parameters may be found in reference [31]. The simulation model was verified by measurements of the time responses for three dynamic states: The step change of the rotor by ± 20 μm in the *x*-axis, the step change of ± 20 μm in the *y*-axis, and the rotor rotation with 4724 rev/min.

**Figure 7:**
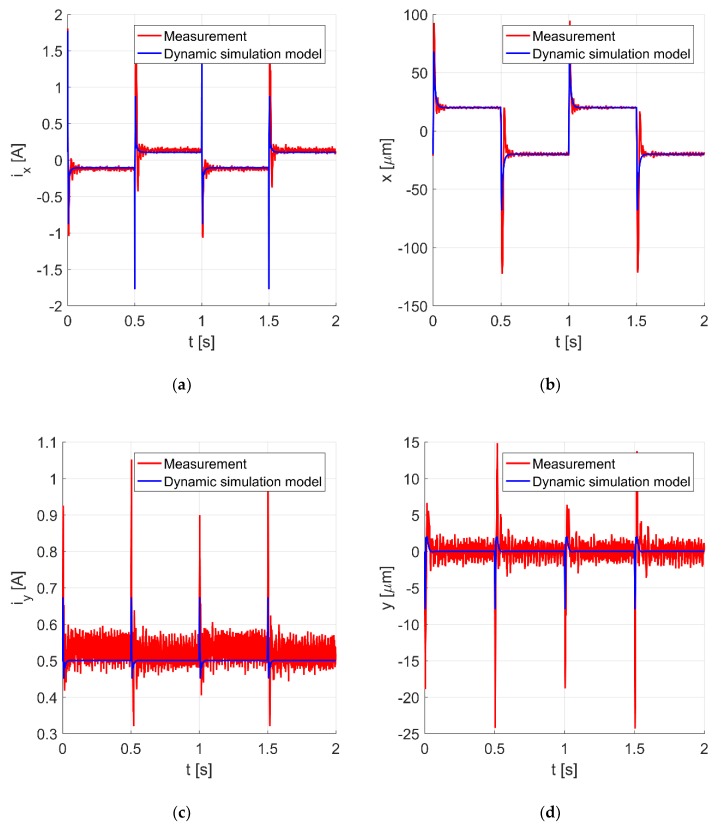
The control current *i_x_* (**a**), the rotor position along the *x*-axis (**b**), the control current *i_y_* (**c**), and the rotor position along the *y*-axis (**d**) for the rotor movement of ± 20 μm in the *x*-axis.

Figure 7a–c and d present the time responses of the control current *i_x_*, the rotor position in the *x*-axis, the control current *i_y,_* and the rotor position in the *y*-axis during the step change of the rotor position along the *x*-axis of ± 20 μm. The noticeable interference signal in the measurements is caused by switching power amplifiers and insufficient electromagnetic shielding of the analog signals.

It can be seen from the measurements and the simulated signals that the change of the rotor position in the *x*-axis caused a change of its position in the *y*-axis. This indicates cross-coupling between axes and points out the correctness of the proposed dynamic simulation model.

**Figure 8:**
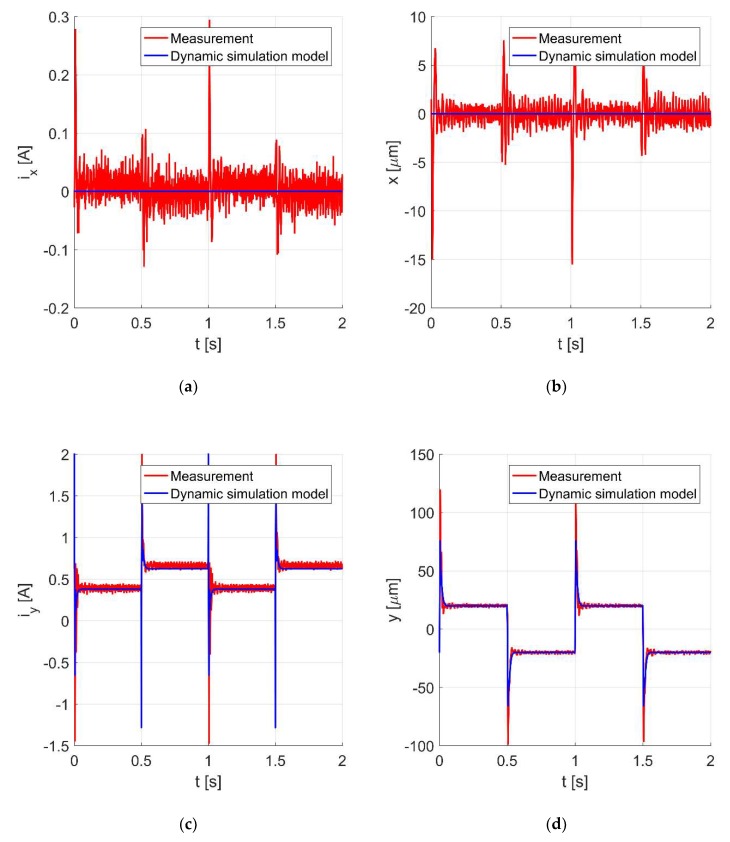
The control current *i_x_* (**a**), the rotor position along the *x*-axis (**b**), the control current *i_y_* (**c**), and the rotor position along the *y*-axis (**d**) for the rotor movement of ± 20 μm in the *y*-axis.

Figure 8a–d present the time responses of the control current *i_x_*, the rotor position *x*, the control current *i_y_*, and the rotor position *y* during the step change of the rotor position along the *y*-axis of ± 20 μm. A disruption of the rotor position in one axis due to the change of the rotor position in the perpendicular axis is clearly visible.

The presented figures indicate a good agreement between the results obtained from the simulations and experiments. The accuracy of the simulation model was assessed by calculating the root mean squared errors (RMSEs) between the measurement and simulation results. The RMSEs were calculated using the following expressions

(15) $$RMSE_{x}=\sqrt{\frac{1}{n}\overset{n}{\underset{k=1}{\sum }}{\left(x_{mes}\left(k\right)-x_{sim}\left(k\right)\right)}^{2}}$$

(16) $$RMSE_{i_{x}}=\sqrt{\frac{1}{n}\overset{n}{\underset{k=1}{\sum }}{\left(i_{x-mes}\left(k\right)-i_{x-sim}\left(k\right)\right)}^{2}}$$

(17) $$RMSE_{y}=\sqrt{\frac{1}{n}\overset{n}{\underset{k=1}{\sum }}{\left(y_{mes}\left(k\right)-y_{sim}\left(k\right)\right)}^{2}}$$

(18) $$RMSE_{i_{y}}=\sqrt{\frac{1}{n}\overset{n}{\underset{k=1}{\sum }}{\left(i_{y-mes}\left(k\right)-i_{y-sim}\left(k\right)\right)}^{2}}$$

where *n* denotes the number of the measurement points. The index *mes* indicates the quantities that were measured, while the index *sim* indicates the quantities that were calculated. RMSEs were calculated for the deviation of ± 20 μm of the rotor along the *x* and *y* axes, respectively, and are shown in Table 5.

**Figure 9:**
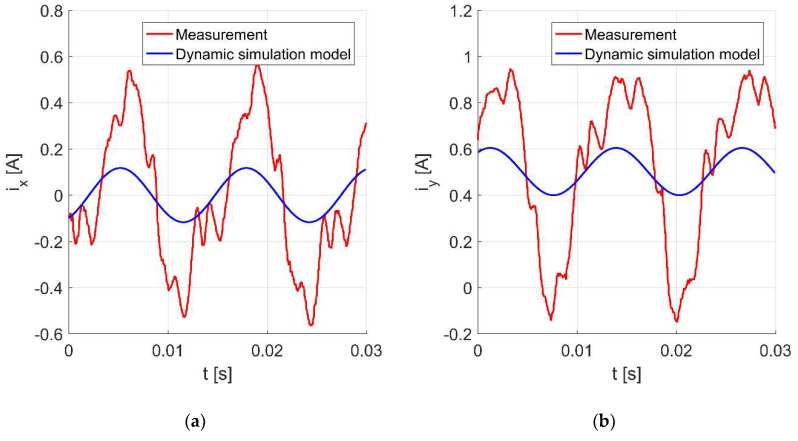
Time responses of the control currents *i_x_* (**a**) and *i_y_* (**b**) for the rotor rotation with 4724 rev/min.

**Figure 10:**
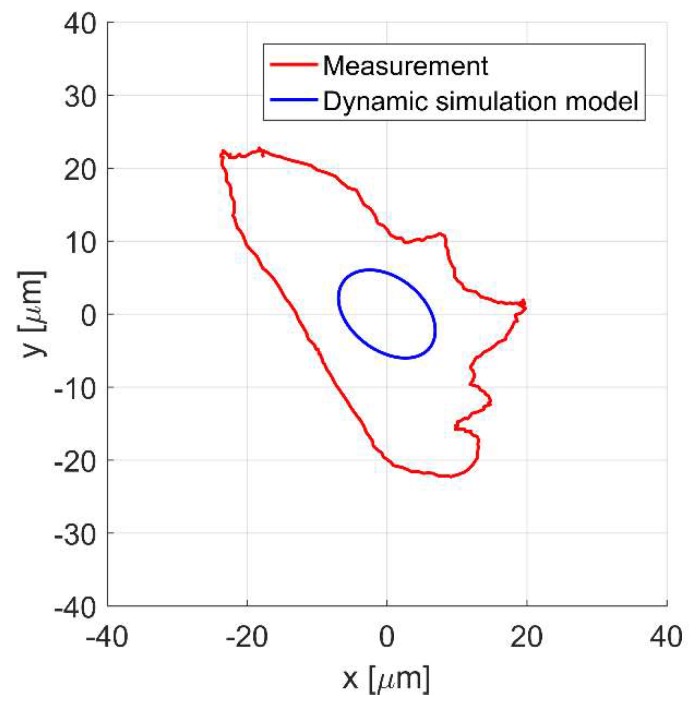
The rotor position in *x* and *y* axes for the rotor rotation with 4724 rev/min.

Figure 9a,b present the time responses of the control currents *i_x_* and *i_y_* for the rotor rotation with 4724 rev/min. The movement of the shaft center is presented in Figure 10. It will be noticed that the values of the control currents obtained from the simulation model are significantly lower than those obtained from the measurement.

Similarly, the rotor displacement obtained from the simulation model is significantly smaller than that obtained from the measurement. Moreover, it can be seen that the trajectory of the rotor movement obtained from the simulation and from the measurement does not create a circle, despite the fact that the static unbalance force varies according to the sine function; this is due to the asymmetrical characteristic of the magnetic force in the *y*-axis [25].

**Figure 11:**
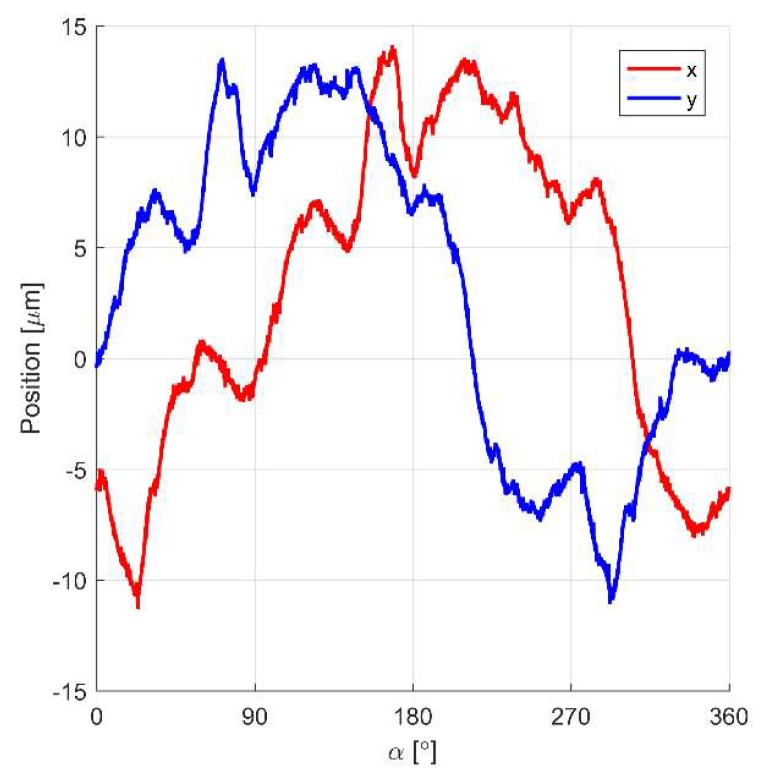
Error signals obtained from the sensors in the *x*- and *y*-axis during slow rotation of the rotor without the control system.

The significant differences between the results obtained from the simulation model and the real object—particularly visible for the shaft rotation—are caused by measurement errors of the rotor position. Figure 11 presents measured errors for the *x*- and *y*-axis in the function of the angular position of the rotor α. Measurements of errors were performed during slow rotation of the rotor set in two ball bearings without the position controllers switched on. Nevertheless, the switching power amplifiers were exciting currents in the windings with the mean value equal to zero. Consequently, the electromagnetic interferences generated by the switching power amplifiers are partially included in the simulation model.

**Figure 12:**
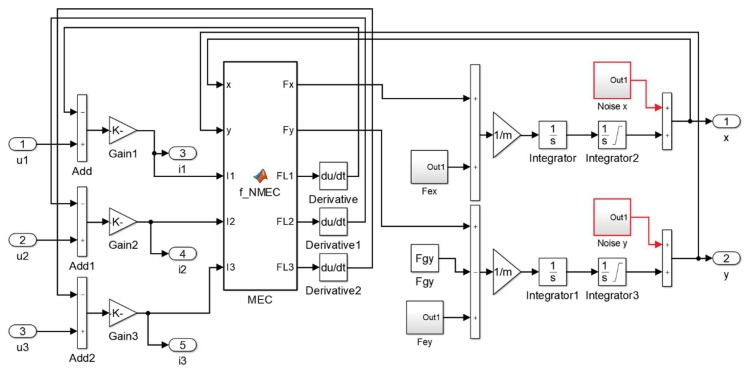
Modified simulation model of the HMB.

The measured errors were incorporated into the simulation model as blocks “Noise x” and “Noise y”. Signals from these blocks are added to the position in the *x* and *y*-axis calculated from the previously presented simulation model. Figure 12 presents a modification of the simulation model indicated by the red color. Under transient magnetic field conditions, a simulation involving the heterogeneous ferromagnetic crystals’ structure of the measurement path for the measurement system would be very time consuming. Moreover, the heterogeneity of the measurement path is unknown. Therefore, the incorporation into the simulation model of the error signals is much more efficient than its simulation.

**Figure 13:**
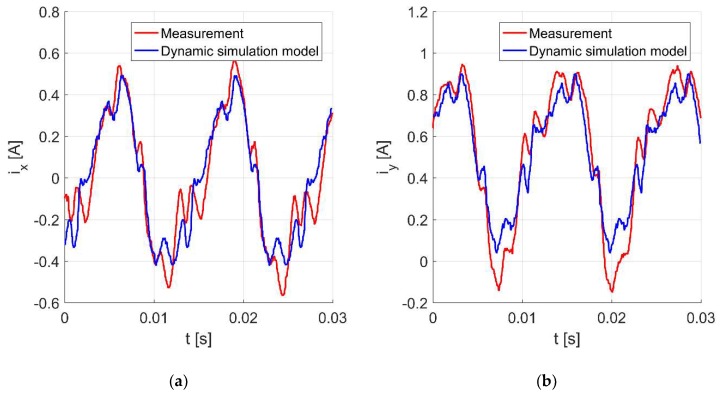
Time responses of the control currents *i_x_* (**a**) and *i_y_* (**b**) for the rotor rotation with 4724 rev/min.

**Figure 14:**
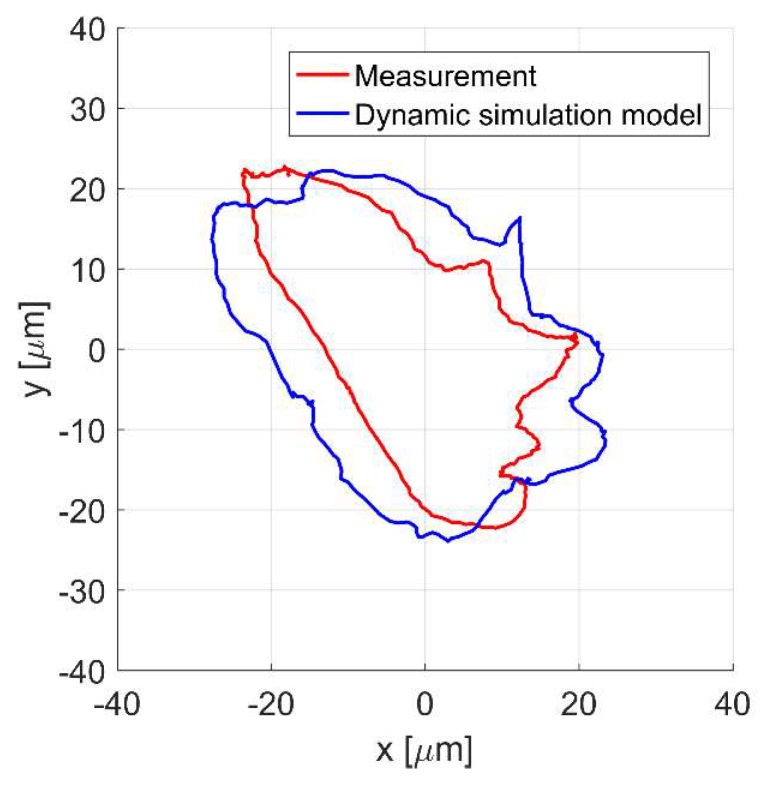
The rotor position in *x* and *y* axes for the rotor rotation with 4724 rev/min.

The next three figures present the time responses of the control currents *i_x_* and *i_y_* Figure 13a,b as well as the displacement of the shaft center for the rotor rotation with 4724 rev/min Figure 14. The improved simulation model factors in errors of the position measurement, and it is noticeable that the response obtained from the simulation model follows more closely the measured one.

## 5. Discussion

The simulations and experiments show that the HMB rotor can levitate stably, and the whole control system yields good dynamics. The paper presents the HMB transients obtained with a field-circuit mathematical model that relies on a magnetic equivalent circuit. The waves of the transients have been compared with the test results. The measurement errors can be caused by a slightly rough cylinder sensor and material heterogeneity of the measurement path. The calculation model has been improved by taking into account the noise signal caused by the measurement system. A good agreement between simulation and experimental results confirms the usefulness of the mathematical model developed in this paper. The proposed methodology significantly reduces the computational time in comparison with a field model that would require the simulation of the eddy currents in the heterogeneous ferromagnetic crystal structure of the sensor path.

## Figures and Tables

**Table 1:** The main parameters of the HMB.

Parameter	Value
Position stiffness *k_sx_*	140.99 N/mm
Current stiffness *k_ix_*	23.22 N/A
Position stiffness *k_sy_*	141.21 N/mm
Current stiffness *k_iy_*	23.16 N/A
Dynamic inductance *L_d_*	4.95 mH
Velocity induced voltage *e_v_*	18.29 Vs/m

**Table 2:** Constant parameters of the simulation model.

Parameter	Value
Winding resistances *R*_1_, *R*_2_, *R*_3_	0.35 Ω
Mass of the rotor reduced to the bearing plane *m*	1.40 kg
Eccentricity *e_s_*	4 μm

**Table 3:** Parameters of the position controllers.

Parameter	Position Controller in the Axis x	Position Controller in the Axis x
*K_P_* [A/m]	8424	8852
*K_I_* [As/m]	120 342	126 500
*K_D_* [A/ms]	13.03	15.26
Sampling time *T_s_* [μs]	100	100
Filter coefficient for derivative *N*	1000	1000

**Table 4:** Parameters of the current controllers.

Parameter	Value
*K_P_* [1/A]	0.35
*K_I_* [1/As]	400
Sampling time *T_s_* [μs]	100

**Table 5:** Root mean squared errors (RMSEs) values for a 2 s transient.

Parameter	The Rotor Movement ±20 μm in the *x*-axis	The Rotor Movement ±20 μm in the *y*-axis
*RMSE_x_*	8.25 μm	1.63 μm
*RMSE_ix_*	124.9 mA	27.0 mA
*RMSE_y_*	1.84 μm	4.27 μm
*RMSE_iy_*	45.3 mA	94.5 mA
